# Primary tumour category, site of metastasis, and baseline serum S100B and LDH are independent prognostic factors for survival in metastatic melanoma patients treated with anti-PD-1

**DOI:** 10.3389/fonc.2023.1237643

**Published:** 2023-08-17

**Authors:** Eszter Anna Janka, Beatrix Ványai, Imre Lőrinc Szabó, Tünde Toka-Farkas, Tünde Várvölgyi, Anikó Kapitány, Andrea Szegedi, Gabriella Emri

**Affiliations:** ^1^ Department of Dermatology, MTA Centre of Excellence, Faculty of Medicine, University of Debrecen, Debrecen, Hungary; ^2^ ELKH-DE Allergology Research Group, Debrecen, Hungary; ^3^ Doctoral School of Health Sciences, University of Debrecen, Debrecen, Hungary

**Keywords:** metastatic melanoma, S100B, LDH, Kaplan-Meier curve, Cox proportional hazard models

## Abstract

**Background:**

Prognostic classification of metastatic melanoma patients treated with anti-PD-1 is of great interest to clinicians.

**Objective:**

We aimed to determine the anti-PD-1 treatment related prognostic performance of demographics, clinical and histological prognostic markers and baseline serum S100B and LDH levels in advanced melanoma.

**Methods:**

A total of 200 patients with unresectable metastatic melanoma were included in this retrospective study. 34.5% had stage M1c disease and 11.5% had stage M1d disease at the start of therapy. 30% had pT4b primary melanoma. 55.5% had elevated baseline serum S100B levels and 62.5% had elevated baseline serum LDH levels. We analysed the risk of death using univariate and multivariate Cox proportional-hazards models and the median overall (OS) and progression-free (PFS) survival using the Kaplan-Meier estimator.

**Results:**

The median follow-up time from the start of anti-PD-1 treatment in patients who were alive at the end of the study (N=81) was 37 months (range: 6.1–95.9). The multivariate Cox regression analysis showed that M1c stage (vs. M1a, p=0.005) or M1d stage at the start of therapy (vs. M1a, p=0.001), pT4b category (vs. pT1a, p=0.036), elevated baseline serum S100B levels (vs. normal S100B, p=0.008) and elevated LDH levels (vs. normal LDH, p=0.049) were independently associated with poor survival. The combination of M1d stage, elevated baseline serum S100B and LDH levels and pT4b category was associated with a very high risk of death (HR 4.72 [1.81; 12.33]). In the subgroup of patients with pT4b primary melanoma, the median OS of patients with normal serum S100B levels was 37.25 months [95% CI 11.04; 63.46]), while the median OS of patients with elevated serum S100B levels was 8.00 months [95% CI 3.49; 12.51]) (p<0.001); the median OS of patients with normal serum LDH levels was 41.82 months [95% CI 11.33; 72.32]), while the median OS of patients with elevated serum LDH levels was 12.29 months [95% CI 4.35; 20.23]) (p=0.002).

**Conclusion:**

Our real-world study indicates that the prognostic role of primary melanoma parameters is preserved in anti-PD-1 treated stage IV patients. Furthermore, there seems to be perspective in combining clinical, histological and serum prognostic markers in a prognostic model.

## Introduction

Cutaneous melanoma prevalence has been increasing in Caucasians for decades (1). It can be mainly explained by increasing incidence of early-stage melanomas, but there is still substantial number of patients with metastatic disease (1, 2). Currently, the standard first-line treatments for advanced melanoma are the immune checkpoint inhibitors and BRAF+ MEK inhibitor combinations (3). Although these agents are much more effective than chemotherapy, treatment failure is quite frequent (3, 4). Identification of biomarkers associated with response to therapy may enhance the treatment effectiveness as well as contributes to better understanding of tumour biology related to melanoma progression (4–7). There is a need for finding well-established and easy access biomarkers (8).

Clinical and pathological prognostic markers for cutaneous melanoma, and molecules associated with tumour growth and metastasis that are released from melanoma cells into the bloodstream during the progression of disease (e.g., S100B, lactate dehydrogenase (LDH), S100A8/A9, soluble PD-L1, matrix metalloproteinase-9, 5-S-cysteinyl-dopa, melanoma inhibiting activity, tyrosinase mRNA, circulating-free DNA BRAFV600E mutation) are potential biomarker candidates (9–19). For prognostic purpose the most reliable biomarker is the American Joint Committee on Cancer (AJCC) TNM classification for melanoma (currently the 8^th^ edition). Categorisation of primary tumours by thickness and ulceration status separates stage IA, IB, IIA, IIB and IIC melanomas with distinct survival outcomes (20). Importantly, the Breslow tumour thickness and ulceration of cutaneous melanoma also have a strong effect on the prognosis of patients with lymphatic metastases, therefore these primary tumour features have been incorporated into the categorisation of stage III melanoma (20). In stage IV the sites of metastases and serum LDH are considered as prognostic markers (20). Of note, pathogenesis of cutaneous melanoma is complex and heterogeneous process that results in primary melanomas with diverse clinicopathological characteristics and prognosis (21). In addition, the activity of molecular pathways associated with melanoma cell growth, resistance to death and invasion is dynamic through the treatment and progression (22). Therefore, it is unlikely that a single biomarker is sufficient to predict disease relapse, progression and response to therapy for all patients. Furthermore, it is likely that the type of treatment has an impact on the prognostic ability of a biomarker (5, 6). Serum markers are expected to be useful for detecting tumour relapse, prognostication as well as prediction of therapeutic response (9, 10). Serum S100B and LDH are thought to correlate with tumour volume and necrosis in metastatic melanoma (23–26). Serum S100B is considered a suitable marker for melanoma recurrence (8), and several studies and reviews have been published on the prognostic effect of serum S100B (27–32). S100B released from melanoma cells is a damage-associated molecular pattern protein that may contribute to tumour-associated inflammation and activate signalling pathways in tumour cells via receptors for advanced glycation end products, thereby promoting melanoma progression (26, 33, 34). A strong correlation between S100B expression in melanoma tumour tissue samples and tumour stage has been found (15, 35, 36). Serum LDH is an established prognostic factor in advanced disease (20). LDH3 and 4 are released from tumour cells dependent on glycolysis (37). Lactate formed during glycolysis is utilized as an energetic source in malignant cells at the more oxygenated tumour periphery as well as may promote angiogenesis, metastasis, therapy resistance, and immunosuppression (37, 38). Serum S100B and LDH levels are routinely monitored in patients with metastatic melanoma in cancer centers.

Few studies have been published so far targeting the prognostic value of traditional prognostic factors used in clinical practice was analysed in a multivariate regression model in advanced melanoma patients (8). The objective of this single-center, retrospective prognostic study was to determine independent prognostic for survival in patients with metastatic melanoma treated with single-agent anti-PD-1. We analysed the risk of death and median overall survival for metastatic melanoma patients treated with anti-PD-1 classifying the patients according to demographics, distant metastasis stage and serum S100B and LDH levels at the beginning of therapy, and clinicopathological features of primary melanoma. Cox univariate and multivariate proportional-hazards models were used to detect and adjust for imbalance in prognostic variables and to estimate a marker-dependent prognosis (39).

## Methods

A total of 200 patients with unresectable metastatic melanoma treated with anti-PD-1 at the Department of Dermatology, Clinical Center, University of Debrecen during the 2015-2022 period were included in this retrospective study. The source of the data was the integrated hospital information system used in the University of Debrecen (MedSolution and UDMED) (Medical Research Council Ethic Committee approved the study; certificate number: IV/1711-4/2021/EKU). The Breslow tumour thickness, ulceration status, localisation, Clark invasion level and histological subtype of primary melanoma, primary tumour (T) stage according to the 8^th^ edition American Joint Committee on Cancer (AJCC) TNM classification for melanoma (20), age and sex of the patient, distant metastasis (M) stage according to the 8^th^ edition AJCC melanoma TNM classification and serum S100B and LDH levels at the start of anti-PD-1 therapy, the anti-PD-1 agent (pembrolizumab or nivolumab), tumour response to anti-PD-1, and patient death were recorded. Serum S100B levels were determined using a quantitative automated chemiluminescent immunoassay (LIAISON® S100). The cut-off point separating normal and elevated serum S100B levels was 0.15 µg/L predefined by the manufacturer. An automated colorimetric assay was used to measure serum LDH levels. The cut-off was the upper limit of the normal LDH level as defined by the local laboratory (220 U/L). Imaging techniques used to evaluate the tumour response were computed tomography (CT), magnetic resonance imaging (MRI) and positron emission tomography with 2-deoxy-2-[fluorine-18]fluoro-D-glucose integrated with computed tomography (18F-FDG PET/CT). Objective response to anti-PD-1 therapy was defined as radiologic complete or partial response according to Response Evaluation Criteria in Solid Tumours (RECIST version 1.1) (40). RECIST version 1.1 was also used to define progressive disease.

Statistical analysis

Overall survival (OS) and progression-free survival (PFS) were analysed using the Kaplan-Meier estimator. OS was calculated from the start of therapy until death by any cause or last moment of follow-up. PFS was calculated from the beginning of therapy until disease progression or the last moment of follow-up. Survival probabilities were compared using a two-sided log-rank test. Median survival time in months was calculated with 95% confidence intervals (95% CI). Hazard ratios (HR) with 95% CI were calculated using Cox regression analysis. The adjustment factors used in the Cox multivariate proportional-hazards model were the following: age and sex of the patient, primary tumour localisation and histological subtype and Clark level, AJCC 8^th^ edition primary melanoma T stage, AJCC 8^th^ edition M stage and serum S100B and LDH levels at initiation of anti-PD-1 therapy, and the anti-PD1 agent. The significance level was set at 0.05 in all cases (∗p<0.05; ∗∗p<0.01; ∗∗∗p<0.001). The data were analysed using IBM SPSS Statistics for Windows, version 25.0 (IBM Corp., Armonk, N.Y., USA).

## Results

### Patient and disease characteristics

200 metastatic melanoma patients treated with anti-PD-1 were included in our study. Due to the efficacy and tolerability shown in clinical trials and real world, and limited access to immune checkpoint inhibitor combinations, in our center most patients with metastatic melanoma are offered anti-PD-1 monotherapy. The clinicopathological characteristics are summarized in **Table 1**. The average age of the patients at the start of anti-PD-1 therapy was 65.58 ± 12.39 years. The Eastern Cooperative Oncology Group performance status (ECOG PS) of the patients was 0 or 1. All patients had Fitzpatrick skin type II or III. 34.5% had stage M1c disease and 11.5% had stage M1d disease at the start of therapy. The median baseline serum S100B level was 0.175 µg/L [inter-quartile range (IQR): 0.07-0.72 µg/L]. The median baseline serum LDH level was 235.5 U/L [IQR: 204.75-301.0 U/L]. The Breslow tumour thickness of the primary melanoma was known in 72% (N=144) of the patients. All patients received single-agent anti-PD-1 therapy, 80% of patients received anti-PD-1 therapy as first line. 23.5% of patients received nivolumab and 76.5% received pembrolizumab. The median overall survival in the entire study population was 15.73 months [IQR: 8.06-36.71 months]. The follow-up time from the start of anti-PD-1 treatment in patients who were alive at the end of the study (N=81) was 6.1–95.9 months (median 37 months).

**Table 1:** Patient and disease characteristics.

Metastatic melanoma patients treated with anti-PD-1	N (%)
Total	200 (100.0)
Age distribution	
<60 years	55 (27.5)
≥60 years	145 (72.5)
Sex	
Male	119 (59.5)
Female	81 (40.5)
Characteristics of primary melanoma in these patients	N (%)
Localisation	
Head and neck	35 (17.5)
Upper limbs	32 (16.0)
Lower limbs	45 (22.5)
Trunk	64 (32.0)
Occult	24 (12.0)
Histological subtype	
SSM	36 (18.0)
LMM	2 (1.0)
ALM	9 (4.5)
NM	76 (38.0)
Mucosal	4 (2.0)
Uveal	1 (0.5)
MM	72 (36.0)
Clark level	
II	4 (2.0)
III	36 (18.0)
IV	66 (33.0)
V	34 (17.0)
unknown	60 (30.0)
AJCC 8^th^ edition T category	
pT1a	6 (3.0)
pT1b-T2a	11 (5.5)
pT2b-T3a	17 (8.5)
pT3b-T4a	39 (19.5)
pT4b	60 (30.0)
Unknown	59 (29.5)
Characteristics of metastatic disease in these patients	
AJCC 8^th^ edition M category at the beginning of anti-PD-1 therapy	
M1a	54 (27.0)
M1b	54 (27.0)
M1c	69 (34.5)
M1d	23 (11.5)
Serum LDH level at the beginning of anti-PD-1 therapy	
normal	75 (37.5)
elevated	125 (62.5)
Serum S100B level at the beginning of anti-PD-1 therapy	
normal	89 (44.5)
elevated	111 (55.5)
Objective response rate to anti-PD-1 therapy	70 (35)
elevated S100B at baseline	31 (27.9)
normal S100B at baseline	39 (43.8)
elevated LDH at baseline	39 (31.2)
normal LDH at baseline	31 (41.3)
Died	119 (59.5)
elevated S100B at baseline	77 (69.4)
normal S100B at baseline	42 (47.2)
elevated LDH at baseline	83 (66.4)
normal LDH at baseline	36 (48.0)

N, number of cases; SSM, superficial spreading melanoma; LMM, lentigo maligna melanoma; ALM, acral lentiginous melanoma; NM, nodular melanoma; MM, unclassified malignant melanoma or no evidence of primary tumour; AJCC, American Joint Committee on Cancer; T, primary tumour; M, distant metastasis.

### Kaplan-Meier survival analysis

In our study, we found no significant differences between patients under 60 years of age and over 60 years of age, or between female and male patients in terms of median OS and median PFS (**Figure S1**).

**Figure 1:**
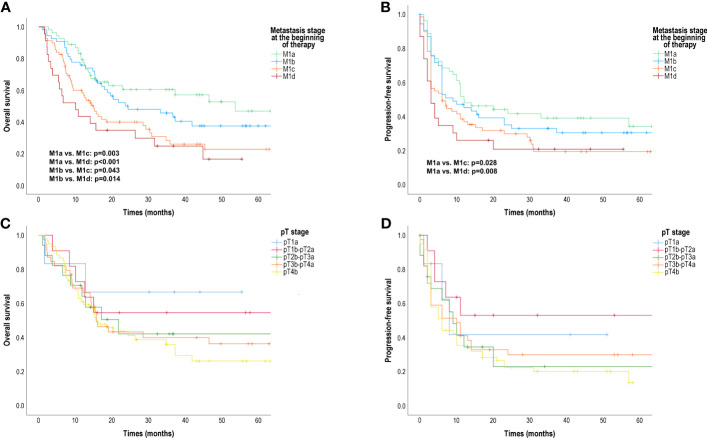
Overall survival (OS) and progression-free survival (PFS) in patients treated with anti-PD-1 according to AJCC 8^th^ edition distant metastasis (M) stage at the start of therapy and primary tumour (pT) category. **(A)** OS according to M stage (months); **(B)** PFS according to M stage (months); **(C)** OS according to pT category (months); **(D)** PFS according to pT category (months); AJCC, American Joint Committee on Cancer Survival probabilities were compared using a two-sided log-rank test.

The median OS of patients with distant metastasis to the central nervous system with or without any other distant sites of disease (M1d) (10.07 months [95% CI 3.03; 17.12]) or non-central nervous system visceral metastases (M1c) (15.00 months [95% CI 11.25; 18.75]) was significantly shorter than the median OS of patients with distant metastasis in the skin, subcutaneous tissue, or distant lymph nodes (M1a) (53.64 months [95% CI 31.87; 75.41]) (M1a vs. M1c: p=0.003; M1a vs. M1d: p<0.001) and/or metastasis to the lung (M1b) (24.36 months [95% CI 6.74; 41.98]) (M1b vs. M1c: p=0.043; M1b vs. M1d: p=0.014) (**Figure 1A**). The median PFS was significantly shorter for patients with M1d (3.00 months [95% CI 1.12; 4.88]) or M1c (6.00 months [95% CI 2.33; 9.67]) than the median PFS for patients with M1a (12.00 months [95% CI 3.84; 20.16]) (M1a vs. M1c: p=0.028; M1a vs. M1d: p=0.008) (**Figure 1B**). There was no significant difference in median OS and median PFS between patients with M1d and patients with M1c.

We found that primary melanoma T category also had an effect on OS and PFS (**Figures 1C, D**). The worst outcome was observed in the case of an ulcerated primary tumour thicker than 4 mm (pT4b). However, the median OS and median PFS of patients with different T stages did not differ significantly.

Furthermore, in the patient population we studied, primary tumour localisation (head and neck, trunk, upper extremities, lower extremities) had no significant effect on OS and PFS (**Figure S2**).

**Figure 2:**
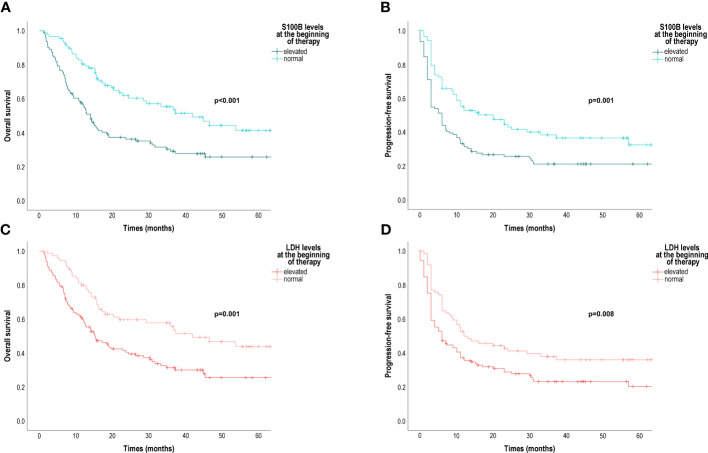
Overall survival (OS) and progression-free survival (PFS) in patients treated with anti-PD-1 according to baseline serum S100B and LDH levels. **(A)** OS according to baseline S100B levels (months); **(B)** PFS according to baseline S100B levels (months); **(C)** OS according to baseline LDH levels (months); **(D)** PFS according to baseline serum LDH levels (months); LDH, lactate dehydrogenase. Survival probabilities were compared using a two-sided log-rank test.

The median OS of patients with normal serum S100B levels was significantly longer (41.82 months [95% CI 27.38; 56.27]) than the median OS of patients with elevated serum S100B levels (14.4 months [95% CI 11.64; 16.44]) (p<0.001) (**Figure 2A**). The median PFS was also significantly longer for patients with normal serum S100B levels (20.00 months [95% CI 7.91; 32.09]) than the median PFS for patients with elevated serum S100B levels (6.00 months [95% CI 4.63; 7.37]) (p=0.001) (**Figure 2B**).

The median OS of patients with normal serum LDH levels was significantly longer (41.82 months [95% CI 22.96; 60.68]) than the median OS of patients with elevated serum LDH levels (15.18 months [95% CI 10.23; 20.13]) (p=0.001) (**Figure 2C**). The median PFS was also significantly longer for patients with normal serum LDH levels (12.00 months [95% CI 2.50; 21.50]) than the median PFS for patients with elevated serum LDH levels (6.00 months [95% CI 3.31; 8.69]) (p=0.008) (**Figure 2D**).

Next, we analysed whether the distant metastasis M stage or the primary melanoma T stage has a prognostic effect in patient subgroups defined according to the level of serum markers (**Figures S3-6**). The median OS was significantly shorter for patients with M1d (6.32 months [95% CI 1.11; 14.63]) or M1c (12.57 months [95% CI 7.07; 18.08]) than the median OS for patients with M1a (37.21 months [95% CI 20.38; 46.89]) in the subgroup of patients with elevated serum S100B (M1a vs. M1c: p=0.036; M1a vs. M1d: p=0.009) (**Figure S3A**). The median OS was significantly shorter for patients with M1d (6.64 months [95% CI 1.12; 14.86]) or M1c (9.39 months [95% CI 4.78; 14.01]) than the median OS for patients with M1a (44.36 months [95% CI 16.16; 62.55]) or M1b (19.61 months [95% CI 6.54; 32.67]) (M1a vs. M1c: p=0.002; M1a vs. M1d: p<0.001; M1b vs. M1c: p=0.049; M1b vs. M1d: p=0.027) in the subgroup of patients with elevated serum LDH (**Figure S4A**).

**Figure 3:**
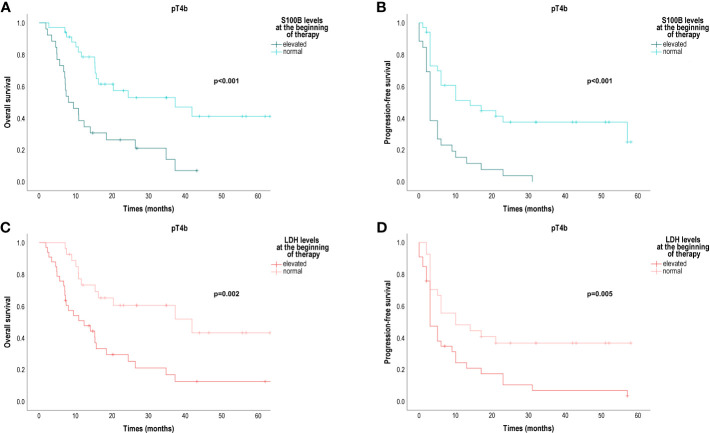
Overall survival (OS) and progression-free survival (PFS) in patients diagnosed with a pT4b primary melanoma and treated with anti-PD-1 for metastatic disease according to baseline serum S100B and LDH levels. **(A)** OS according to baseline S100B levels (months); **(B)** PFS according to baseline S100B levels (months); **(C)** OS according to baseline LDH levels (months); **(D)** PFS according to baseline serum LDH levels (months); LDH, lactate dehydrogenase. Survival probabilities were compared using a two-sided log-rank test.

Importantly, the prognostic value of serum S100B and LDH levels seemed to be very pronounced in patients diagnosed with a pT4b primary melanoma. In this group of patients, the median OS of patients with normal serum S100B levels was 37.25 months [95% CI 11.04; 63.46]), while the median OS of patients with elevated serum S100B levels was 8.00 months [95% CI 3.49; 12.51]) (p<0.001) (**Figure 3A**). The median PFS for patients with normal serum S100B levels was 14.00 months [95% CI 2.14; 25.86]), while the median PFS for patients with elevated serum S100B levels was 3.00 months [95% CI 2.39; 3.61]) (p<0.001) (**Figure 3B**). The median OS of patients with normal serum LDH levels was 41.82 months [95% CI 11.33; 72.32]), while the median OS of patients with elevated serum LDH levels was 12.29 months [95% CI 4.35; 20.23]) (p=0.002) (**Figure 3C**). The median PFS for patients with normal serum LDH levels was 10.79 months [95% CI 1.61; 20.96]), while the median PFS for patients with elevated serum LDH levels was 3.00 months [95% CI 1.60; 4.41]) (p=0.005) (**Figure 3D**).

### Cox regression analysis

**Figure 4:**
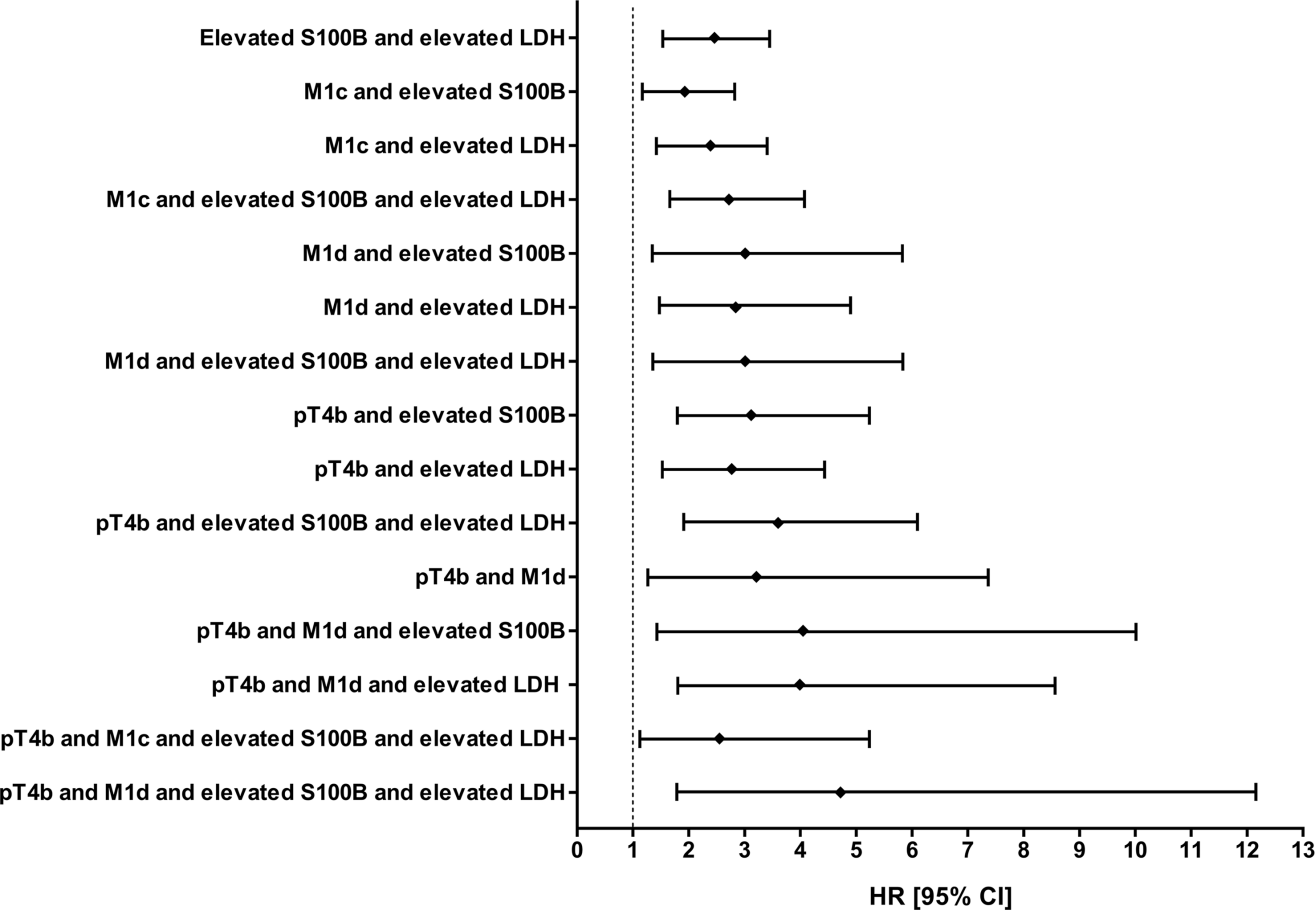
Significant results from multivariate Cox proportional-hazards models considering combinations of independent prognostic factors for survival in patients with metastatic melanoma treated with anti-PD-1. HR [95% CI], hazard ratios with 95% confidence intervals.

Cox multivariate proportional-hazards models of overall survival were used to determine the independence of prognostic factors. In our study, M1d stage (HR 3.43 [95% CI 1.70; 6.89] vs. M1a stage), M1c stage (HR 2.17 [95% CI 1.27; 3.72] vs. M1a stage), pT4b stage (HR 2.77 [95% CI 1.07; 7.19] vs. T1a stage), elevated serum S100B levels (HR 1.87 [95% CI 1.17; 2.99] vs. normal S100B) and elevated LDH levels (HR 1.57 [95% CI 1.01; 2.45] vs. normal LDH) independently were associated with a risk of death in advanced melanoma patients treated with anti-PD-1 (**Table 2**). The prognostic performance of combinations of these factors was also analysed in Cox univariate and multivariate proportional-hazards models (**Table 3**). Our data showed that the risk of death was very high in patients with elevated levels of both serum markers and those with elevated serum marker levels and pT4b primary melanoma stage and/or M1d distant metastasis stage (**Table 3**, **Figure 4**).

**Table 2:** Independent determinants of survival in patients with metastatic melanoma treated with anti-PD-1 by Cox proportional-hazards model.

Variables	Subcategories	Multivariate Cox model	
		HR [95% CI]	p-value
AJCC 8^th^ edition M category at the beginning of therapy	M1c/M1a	**2.17 [1.27; 3.72]**	**0.005**
	M1d/M1a	**3.43 [1.70; 6.89]**	**0.001**
AJCC 8^th^ edition T category	pT4b/pT1a	**2.77 [1.07; 7.19]**	**0.036**
Serum S100B level at the beginning of therapy	elevated/normal	**1.87 [1.17; 2.99]**	**0.008**
Serum LDH level at the beginning of therapy	elevated/normal	**1.57 [1.01; 2.45]**	**0.049**

The adjustment factors used in the multivariate model were the following: age and sex of the patient, primary tumour localisation and histological subtype and Clark level and pathological stage, distant metastasis stage and serum S100B and LDH levels at the beginning of anti-PD-1 therapy, and the anti-PD1 agent. Only the significant results are shown in this Table.

HR [95% CI], hazard ratio with 95% confidence intervals; AJCC, American Joint Committee on Cancer; T, primary tumour; M, distant metastasis.

Significant results are in bold.

**Table 3:** Univariate and multivariate Cox proportional-hazards models considering combinations of independent prognostic factors for survival in patients with metastatic melanoma treated with anti-PD-1 .

Combined variables	Univariate Cox model		Multivariate Cox model	
	HR [95% CI]	p-value	HR [95% CI]	p-value
Elevated S100B and elevated LDH	**2.28 [1.59; 3.28]**	**<0.001**	**2.46 [1.68; 3.61]**	**<0.001**
M1c and elevated S100B	**1.75 [1.19; 2.57]**	**0.004**	**1.93 [1.27; 2.94]**	**0.002**
M1c and elevated LDH	**2.10 [1.42; 3.10]**	**<0.001**	**2.39 [1.57; 3.64]**	**<0.001**
M1c and elevated S100B and elevated LDH	**2.25 [1.51; 3.35]**	**<0.001**	**2.72 [1.75; 4.20]**	**<0.001**
M1d and elevated S100B	**2.49 [1.34; 4.65]**	**0.004**	**3.01 [1.52; 5.95]**	**0.002**
M1d and elevated LDH	**2.41 [1.44; 4.04]**	**0.001**	**2.84 [1.61; 5.00]**	**<0.001**
M1d and elevated S100B and elevated LDH	**2.49 [1.34; 4.66]**	**0.004**	**3.01 [1.52; 5.95]**	**0.002**
pT4b and elevated S100B	**2.46 [1.54; 3.94]**	**<0.001**	**3.12 [1.84; 5.30]**	**<0.001**
pT4b and elevated LDH	**2.13 [1.38; 3.28]**	**0.001**	**2.77 [1.66; 4.62]**	**<0.001**
pT4b and elevated S100B and elevated LDH	**2.80 [1.72; 4.56]**	**<0.001**	**3.60 [2.08; 6.23]**	**<0.001**
pT4b and M1c	1.46 [0.80; 2.66]	0.219	–	–
pT4b and M1c and elevated S100B	1.51 [0.76; 2.98]	0.238	–	–
pT4b and M1c and elevated LDH	1.78 [0.93; 3.42]	0.084	–	–
pT4b and M1c and elevated S100B and elevated LDH	**2.02 [1.02; 4.00]**	**0.044**	**2.55 [1.22; 5.30]**	**0.012**
pT4b and M1d	**2.34 [1.09; 5.03]**	**0.029**	**3.21 [1.37; 7.50]**	**0.007**
pT4b and M1d and elevated S100B	**3.76 [1.53; 9.25]**	**0.004**	**4.05 [1.62; 10.13]**	**0.003**
pT4b and M1d and elevated LDH	**3.65 [1.69; 7.88]**	**0.001**	**3.99 [1.81; 8.77]**	**0.001**
pT4b and M1d and elevated S100B and elevated LDH	**3.76 [1.53; 9.25]**	**0.004**	**4.72 [1.81; 12.33]**	**0.002**

The adjustment factors used in the multivariate model were the following: age and sex of the patient, primary tumour localization and histological subtype and Clark level and pathological stage, distant metastasis stage and serum S100B and LDH levels at the beginning of anti-PD-1 therapy, and the anti-PD1 agent. Significant results are in bold.

HR [95% CI], hazard ratios with 95% confidence intervals; pT4b, via AJCC 8^th^ edition primary tumour category, pT4b: tumour thickness >4.0 mm with ulceration; M1c and M1d – AJCC 8^th^ edition distant metastasis categories, M1c: patients with non-central nervous system visceral metastases, M1d: patients with distant metastasis to the central nervous system with or without any other distant sites of disease.

## Discussion

The thickness and ulceration status of the primary tumour are the strongest predictors of metastasis formation and thus survival at the time of diagnosis in melanoma (20). Interestingly, melanoma-specific survival also depends on the primary tumour in patients with regional lymph node metastases (20). The 8^th^ edition AJCC TNM classification indicates a prognostic difference between N3a-c metastatic cases in stage III melanoma according to whether the primary tumour was pT4b or not (IIID vs. IIIC). In stage IV, the risk of death is strongly influenced by the anatomic site of distant metastases and serum LDH levels (41). The clinical outcome is difficult to predict in the case of metastatic melanoma treated with immunotherapy, and a more accurate prognostic classification is of great interest to clinicians. In this retrospective study the prognostic performance of demographic data, clinical and histological prognostic markers and baseline serum LDH and S100B levels were tested in metastatic melanoma patients treated with anti-PD-1 using Cox regression and Kaplan-Meier survival analyses. The relatively high number of patients enabled subgroup analysis, and the relatively long follow-up time allowed a more accurate estimate of the risk of death.

Age and sex influence the incidence and prognosis of primary melanoma (2, 42), so we would expect a difference in the survival of patients with metastatic melanoma according to age and sex. However, in the present study, we found no significant difference in the survival of patients under 60 years of age and over 60 years of age, or in the survival of female and male patients. Similarly, Zhao et al., in a study using logistic regression, found that age and sex did not affect the survival of patients treated with anti- PD-1 (43). Additionally, in a recently reported population-based cohort study that analysed data from more than 1,000 melanoma patients aged 65 years and older, there was no significant difference in survival between male and female patients receiving anti-PD-1 therapy (44). Of note, a study evaluating factors influencing tumour response to anti-PD-1 therapy found that response to therapy was lower among those younger than 65 years and among women (45). In another study aimed at identifying factors associated with the development of primary resistance to immune checkpoint inhibitor therapy, age and sex were not significant factors in multivariate logistic regression analysis (46). Further studies are needed to explore direct and indirect age- and sex-related factors influencing tumour progression and anti-tumour immunity.

Our data confirmed that the site of distant metastases significantly affects disease outcome. In patients treated with anti-PD-1, the prognosis was worse if there were also brain metastases or visceral metastases than if there were only skin/distant lymph nodes metastases. In certain metastatic sites (e.g., liver, bone, brain), tumour cells are more likely to evade immune surveillance (47). In addition, high tumour burden is often associated with stage M1c or M1d disease. In studies analysing the independence of prognostic factors, various parameters related to the site of metastasis and tumour burden are included, e.g., elevated baseline serum LDH levels, M stage, the presence of liver metastases, the presence of brain metastases (43, 45, 46, 48). Recently, a new prognostic risk model has been proposed for advanced melanoma patients treated with immune checkpoint inhibitors (49). The presence of liver metastases, ECOG PS≥1, elevated serum LDH levels and markers of systemic inflammatory burden (hypoalbuminemia, elevated white blood cell count) were included in the model (49). These studies support that readily available clinical parameters can be combined into a prognostic model.

We found that the primary tumour T category remains an independent prognostic factor even in the case of stage IV. This underscores the importance of primary tumour features in biological behaviour of melanoma in advanced disease. The fact that primary melanoma can affect the outcome of metastatic melanoma, has also been suggested by other studies. For example, primary tumour localisation was identified as an independent prognostic factor for overall survival in stage IV melanoma patients treated with first-line anti-PD-1-based therapy (46). Nodular melanoma histological subtype was found to be an independent risk factor for death in metastatic melanoma patients treated with BRAF- ± MEK-inhibitors but not in those treated with immunotherapy (50). However, in another study, the nodular melanoma subtype did not prove to be an independent prognostic factor in advanced melanoma patients treated with BRAF- ± MEK-inhibitors or immunotherapy (51). To the best of our knowledge, this is the first report that primary melanoma pT4b category is an independent predictor of mortality in stage IV melanoma patients treated with anti-PD-1. Whether molecular markers related to immunotherapy efficacy, such as tumour mutational burden and inflammatory gene expression (5), differ in metastases originating from pT4b primary tumours and those originating from non-pT4b primary tumours requires further investigation. Driver mutations responsible for tumour development, as well as cumulative sun damage, can be important influencing factors of both the clinicopathological characteristics of the primary tumour and the responsiveness to immunotherapy (52). Of note, the median overall survival times according to the primary melanoma T category did not differ significantly, the separation of the Kaplan-Meier curves appears at longer follow-up times.

Serum biomarkers may provide relevant information on melanoma patient status. In a meta-analysis, we previously found that both serum S100B and serum LDH are valuable prognostic markers in advanced melanoma patients (8). Serum S100B is more specific for melanoma than serum LDH, however, serum S100B levels can be elevated in many other diseases, such as neurodegenerative diseases, previous stroke, migraine, acute brain injury, inflammatory bowel disease, liver cirrhosis, diabetes, cardiovascular diseases (8, 53), thus comorbidities may affect the prognostic value of serum S100B in patients with metastatic melanoma. Serum S100B levels can also be influenced by skin pigmentation (54). All patients in our study were Caucasian. It is not known whether melanin production in melanoma cells affected serum S100B levels. Serum S100B is also a marker of brain injury (53), and there is a high chance that serum S100B levels are elevated in melanoma patients with central nervous system metastases. Importantly, the active role of extracellular S100B in neuroinflammation was revealed, and through similar processes, S100B may play a role in the progression of metastatic melanoma, contributing to the unfavourable outcome of the disease (53). In retrospective studies performed among metastatic melanoma patients treated with anti-PD-1, anti-CTLA-4 or anti-CTLA-4+anti-PD-1 the baseline serum S100B level proved to be independent predictor of primary resistance to therapy and overall survival (46, 48, 55). In addition, the change in serum S100B levels during the first weeks of immune checkpoint inhibitor therapy appeared associated with therapeutic response and overall survival (48, 56). Predictive performance of baseline serum LDH or early change in serum LDH levels seemed to be limited (46, 48, 55, 56). In our study, we found significantly better survival data for normal baseline serum S100B and LDH concentrations than for elevated baseline serum S100B and LDH levels. In multivariate analysis serum S100B and LDH were identified as independent prognostic factors for survival.

It is noteworthy that combination of elevated serum S100B and Breslow tumour thickness of >4 mm increased the diagnostic accuracy for detecting metastatic disease in melanoma (57). In our study, the difference in median OS and median PFS between patients with normal serum S100B levels and those with elevated serum S100B levels was very pronounced in patients with pT4b primary melanoma . Also, the difference in median OS and median PFS between patients with normal serum LDH levels and those with elevated serum LDH levels was very pronounced in patients with pT4b primary melanoma. We analysed combinations of independent prognostic factors in univariate and multivariate Cox proportional-hazards models. We found that different combinations of these factors were associated with different hazard ratios. Thus, these prognostic factors, i.e., clinicopathological features of the primary melanoma, anatomic site of distant metastases, and baseline serum S100B and LDH levels are good candidates for a multivariable prognostic model, but validation is needed.

The strength of this study was the relatively high number of patients, the relatively long follow-up time, and the use of Cox regression analysis. Baseline serum S100B and LDH levels were available for all patients. Limitations: It was a single-center retrospective analysis, and the prognostic model could not yet be validated. Subgroup analysis was limited by the number of cases. Data on primary tumours were available in many cases, but not in all cases. Metastatic melanoma patients receiving anti-PD-1 treatment at our center were included in the study without selection, however, it was a single center study that may have introduced selection bias. For example, the majority of the patients had elevated baseline serum LDH levels and pT4b primaries, indicating that the patients in this cohort had a poor prognosis. Analysing the prognostic power of the combination of 3 or 4 prognostic factors in Cox proportional-hazards models, the results were significant, but, due to the stratification, the 95% confidence intervals are wide, which means that further analysis is needed on a larger group of patients.

## Conclusion

Our study suggests that primary melanoma parameters have a prognostic role even in stage IV melanoma patients, at least in case of anti-PD-1 treatment. It highlights the need for further research into the biology of primary melanoma and micrometastatic disease. Furthermore, research on metastatic melanoma with elevated serum S100B and LDH levels may provide a step forward to improve treatment efficiency.

## Author contributions

Concept and design: EJ, BV, GE. Acquisition, analysis, or interpretation of data: EJ BV, GE. Statistical analysis was performed by a biostatistician: EJ. Drafting of the manuscript: EJ, BV, GE. Critical revision of the manuscript: all authors. All of the co-authors granted final approval of the version of the article to be published.
