# Supplementary material for: Primary tumour category, site of metastasis, and baseline serum S100B and LDH are independent prognostic factors for survival in metastatic melanoma patients treated with anti-PD-1
Source: Front Oncol. 2023 Aug 17;13:1237643. doi: 10.3389/fonc.2023.1237643 (PMC10472446; doi:10.3389/fonc.2023.1237643)
Supplement: Supplementary file 1 [file DataSheet_1.pdf]

Supplementary Material

Supplementary Figures

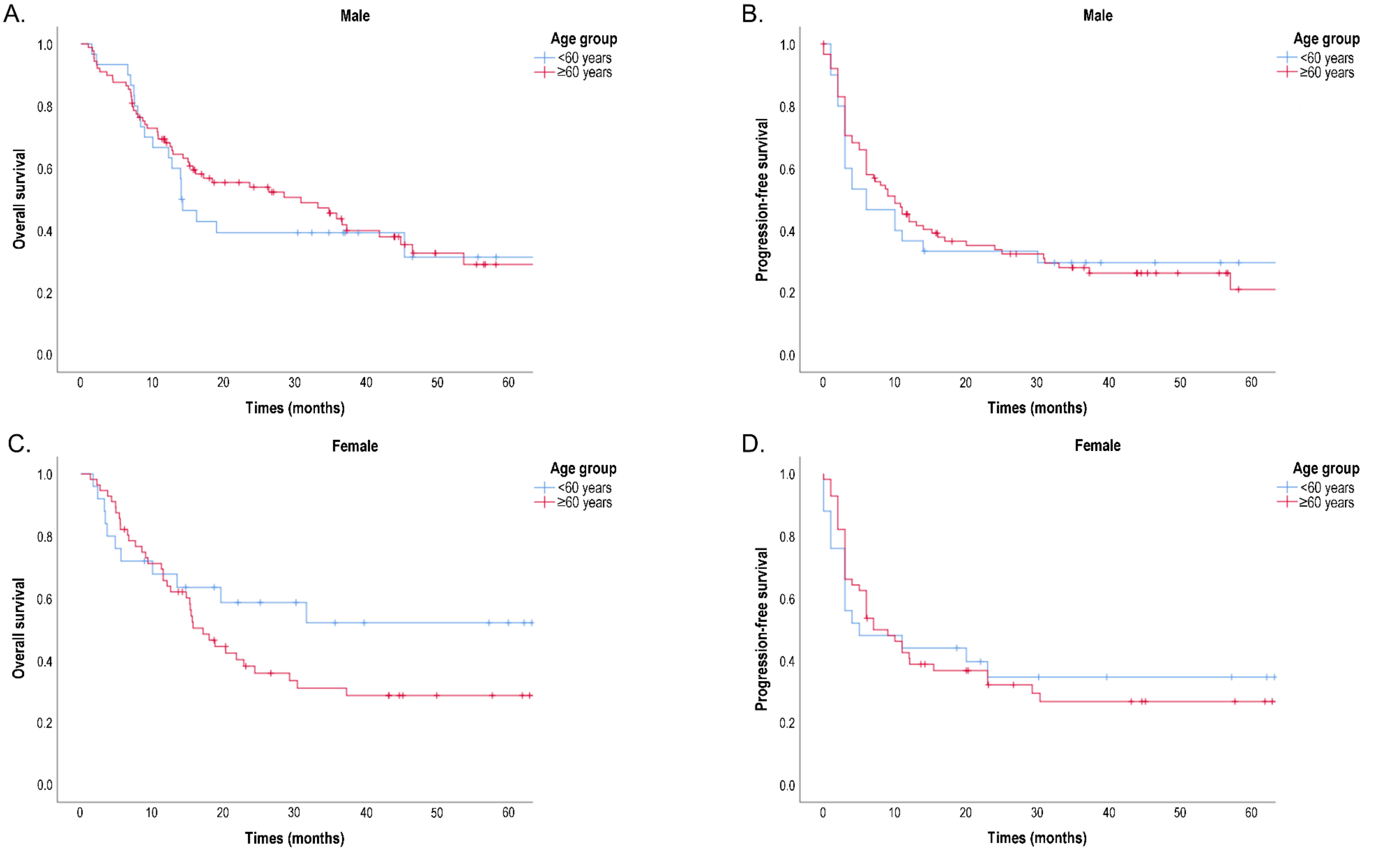

Supplementary Figure 1. Overall survival (OS) and progression-free survival (PFS) in patients treated with anti-PD-1 by age group (male patients versus female patients)

A. OS by age group in male patients (months); B. PFS by age group in male patients (months); C. OS by age group in female patients (months); D. PFS by age group in female patients (months). Survival probabilities were compared using a two-sided log-rank test.

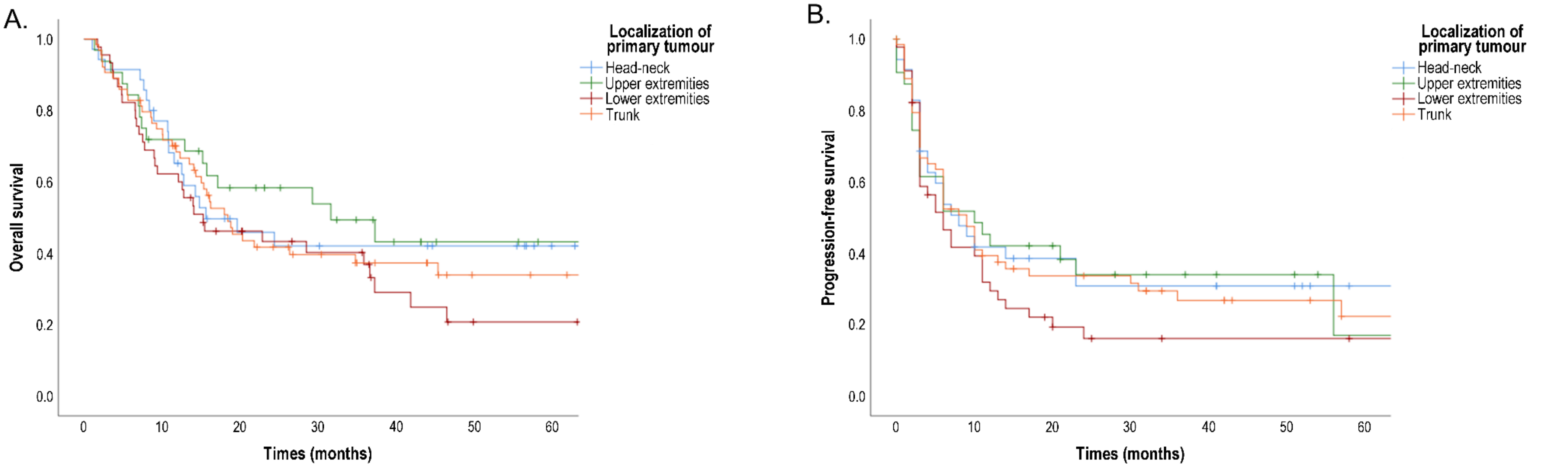

Supplementary Figure 2. Overall survival (OS) and progression-free survival (PFS) in patients treated with anti-PD-1 according to primary tumour localization

A. OS according to primary tumour localization (months); B. PFS according to primary tumour localization (months). Survival probabilities were compared using a two-sided log-rank test.

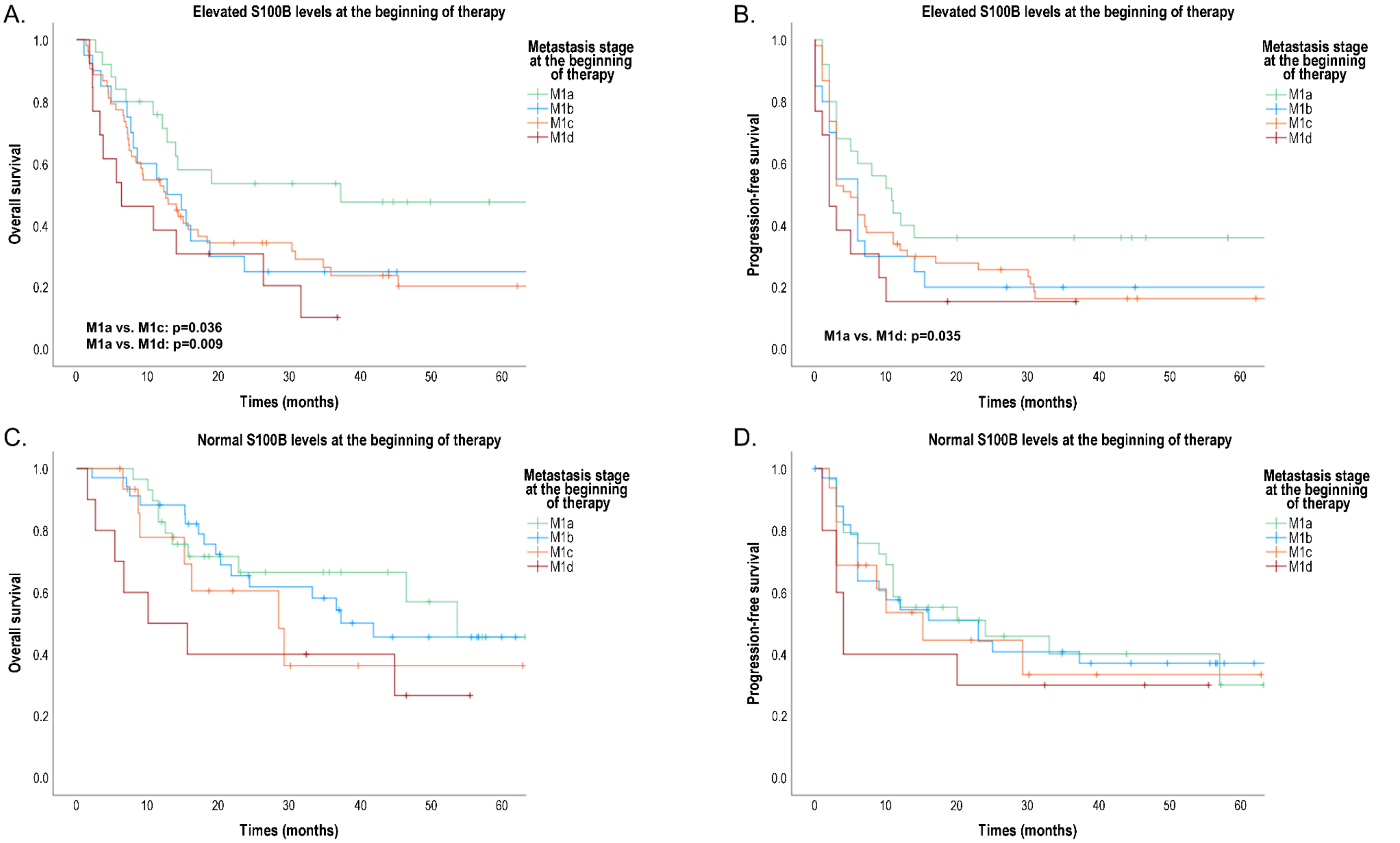

Supplementary Figure 3. Overall survival (OS) and progression-free survival (PFS) in patients treated with anti-PD-1 according to AJCC 8th edition distant metastasis (M) stage at the start of therapy (elevated baseline serum S100B levels versus normal baseline serum S100B levels)

A. OS in patients with elevated baseline serum S100B levels according to M stage (months); B. PFS in patients with elevated baseline serum S100B levels according to M stage (months); C. OS in patients with normal baseline serum S100B levels according to M stage (months); D. PFS in patients with normal baseline serum S100B levels according to M stage (months). Survival probabilities were compared using a two-sided log-rank test.
AJCC – American Joint Committee on Cancer

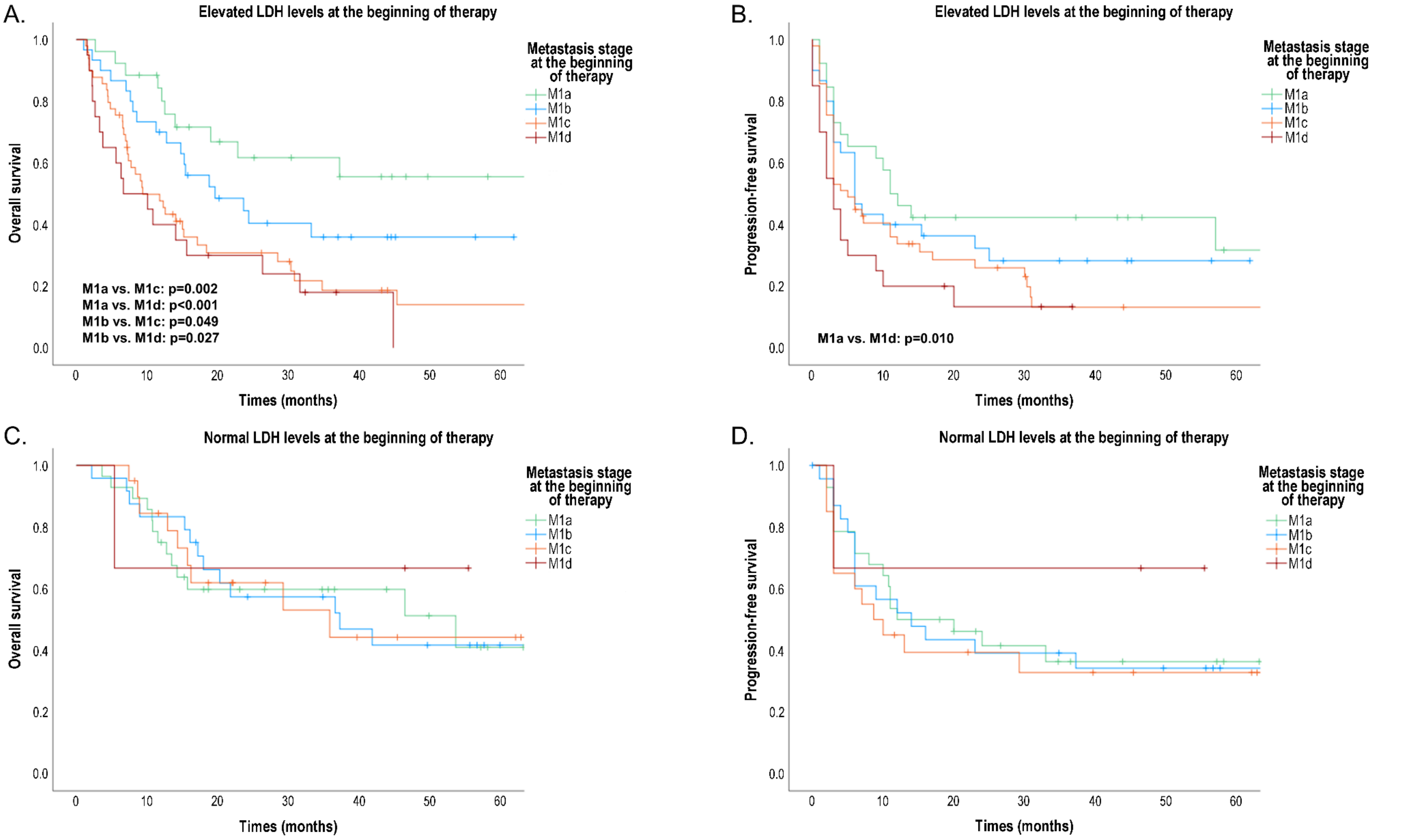

Supplementary Figure 4. Overall survival (OS) and progression-free survival (PFS) in patients treated with anti-PD-1 according to AJCC 8th edition distant metastasis (M) stage at the start of therapy (elevated baseline serum LDH levels versus normal baseline serum LDH levels)

A. OS in patients with elevated baseline serum LDH levels according to M stage (months); B. PFS in patients with elevated baseline serum LDH levels according to M stage (months); C. OS in patients with normal baseline serum LDH levels according to M stage (months); D. PFS

in patients with normal baseline serum LDH levels according to M stage (months). Survival probabilities were compared using a two-sided log-rank test.
AJCC – American Joint Committee on Cancer; LDH – lactate dehydrogenase

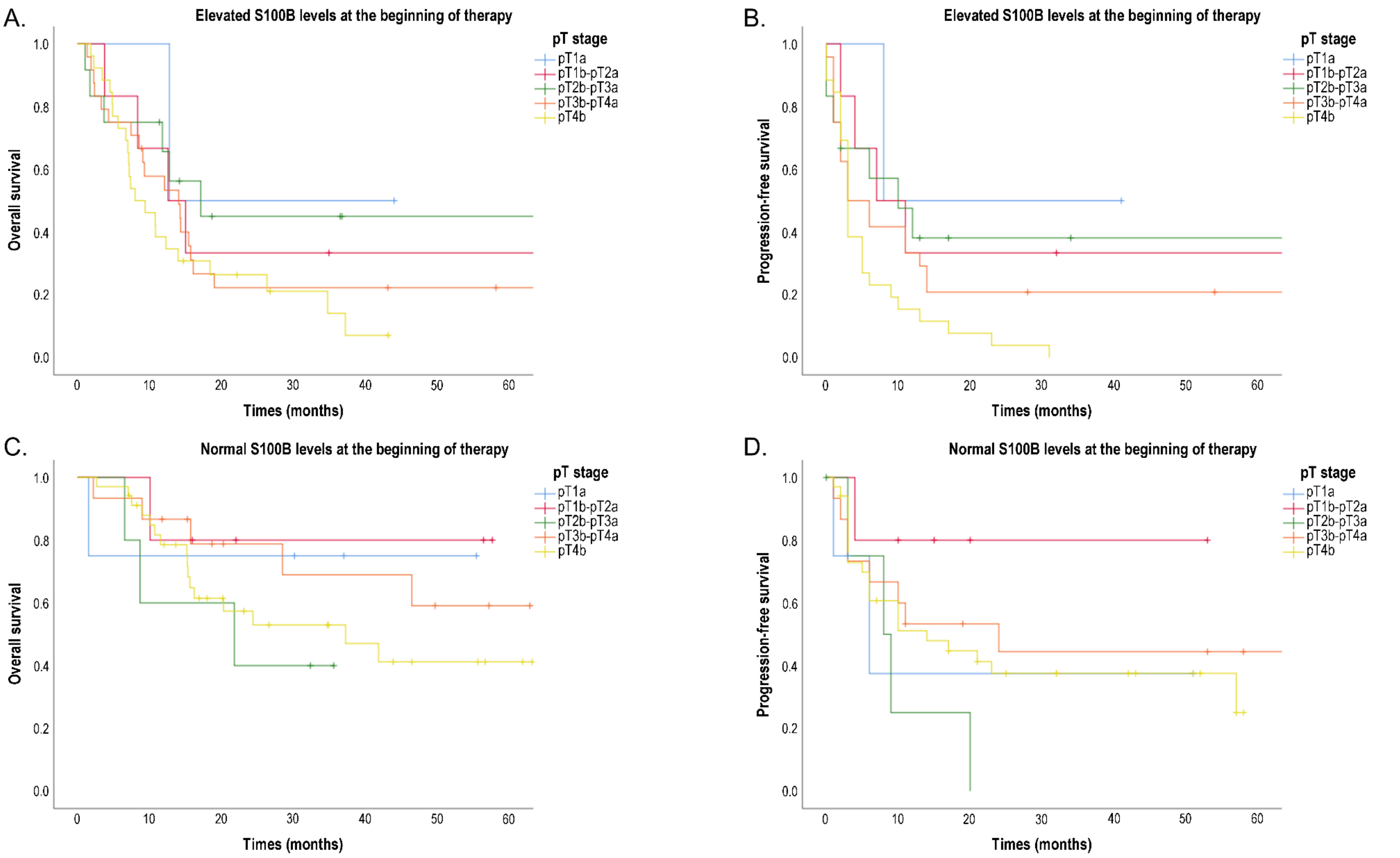

Supplementary Figure 5. Overall survival (OS) and progression-free survival (PFS) in patients treated with anti-PD-1 according to AJCC 8th edition primary tumour (pT) category (elevated baseline serum S100B levels versus normal baseline serum S100B levels)
A. OS in patients with elevated baseline serum S100B levels according to pT category (months); B. PFS in patients with elevated baseline serum S100B levels according to pT category (months); C. OS in patients with normal baseline serum S100B levels according to pT category

(months); D. PFS in patients with normal baseline serum S100B levels according to pT category (months). Survival probabilities were compared using a two-sided log-rank test.
AJCC – American Joint Committee on Cancer

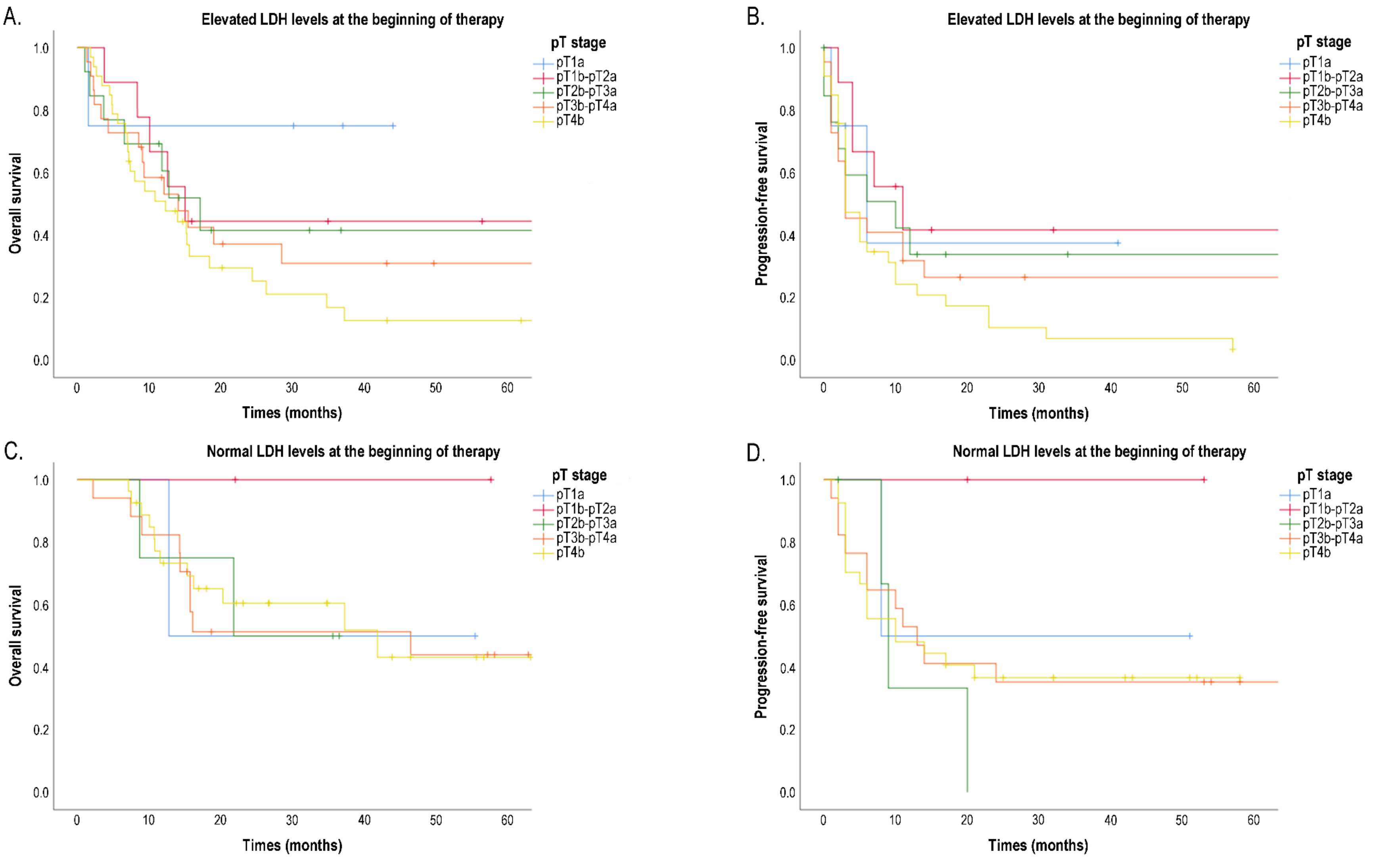

Supplementary Figure 6. Overall survival (OS) and progression-free survival (PFS) in patients treated with anti-PD-1 according to AJCC 8th edition primary tumour (pT) category (elevated baseline serum LDH levels versus normal baseline serum LDH levels)
A. OS in patients with elevated baseline serum LDH levels according to pT category (months); B. PFS in patients with elevated baseline serum LDH levels according to pT category (months); C. OS in patients with normal baseline serum LDH levels according to pT category (months);

D. PFS in patients with normal baseline serum LDH levels according to pT category (months). Survival probabilities were compared using a two-sided log-rank test.
AJCC – American Joint Committee on Cancer; LDH – lactate dehydrogenase
